# A case report of a rare and distinctive pacing pattern during left bundle branch pacing in a child with third-degree atrioventricular block following ventricular septal defect repair

**DOI:** 10.3389/fcvm.2026.1826553

**Published:** 2026-06-15

**Authors:** Yue Bao, Wenwen Chen, Qingquan Zhang, Hongwei Han

**Affiliations:** Department of Cardiology, Wuhan Asia Heart Hospital, Wuhan, China

**Keywords:** left bundle branch pacing, pacing electrocardiogram, right bundle branch block, third-degree atrioventricular block, ventricular septal defect repair

## Abstract

After repair of a ventricular septal defect (VSD), atrioventricular conduction block frequently occurs, often requiring pacemaker implantation. Left bundle branch pacing (LBBP) is a physiological pacing modality and has become the preferred pacing approach in pacemaker-dependent children. Conventional right ventricular septal pacing typically produces a left bundle branch block (LBBB) pattern on the electrocardiogram (ECG). Herein, we report the case of a 3-year-old child who developed third-degree atrioventricular block complicated by right bundle branch block (RBBB) after VSD repair. The child subsequently received LBBP. During the procedure, the pacing lead only made superficial contact with the right ventricular septal myocardium without deep septal fixation, yielding an RBBB pattern on ECG. No relevant similar cases have been identified in the current literature, rendering this phenomenon rare in clinical practice.

## Introduction

Atrioventricular block occurring after VSD repair is one of the common complications (1) and often necessitates permanent pacemaker implantation. LBBP directly activates the His–Purkinje system to preserve normal ventricular depolarization sequence, thereby avoiding the adverse effects of ventricular dyssynchrony; it has thus become the preferred pacing modality for patients with ventricular pacing dependence (2). This procedure involves advancing the lead through the interventricular septum to reach the left bundle branch region. In the early stage of implantation, when the lead initially contacts the right ventricular septal myocardium, temporary right ventricular septal pacing is achieved, and the ECG typically demonstrates an LBBB pattern (3, 4). However, in the case, an RBBB pattern appeared unexpectedly when the lead was placed in contact with the right ventricular septal myocardium. This phenomenon is extremely rare. Previous studies have suggested that an RBBB pattern during initial pacing is usually associated with procedural complications, such as lead displacement or myocardial perforation, rather than physiological conduction system pacing. Moreover, the incidence of pacing-induced RBBB is significantly higher with right ventricular apical pacing than with septal pacing. Importantly, this pacing-induced RBBB pattern is unrelated to the presence of underlying RBBB in the baseline sinus rhythm (5, 6). This phenomenon may be related to proximal conduction system block and surgical scarring.

## Case report

**Figure 1:**
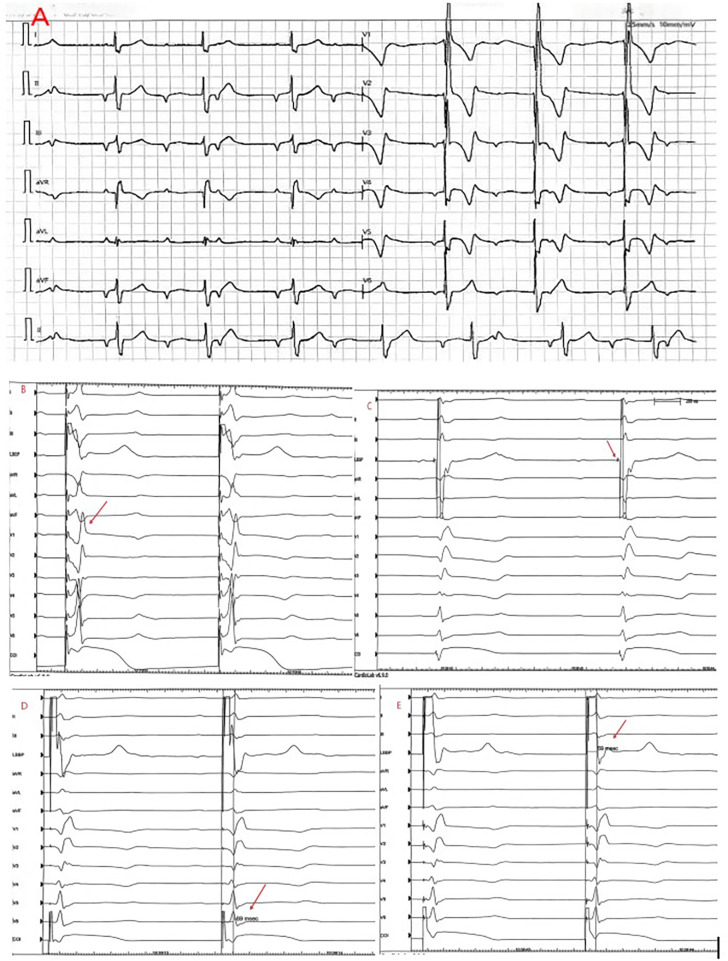
(A) ECG showing third-degree atrioventricular block and RBBB. (B) Pacing morphology showing an rsR′ pattern when the lead contacted the right ventricular septal surface. (C) A left bundle branch potential was observed after rotating the electrode to the left side of the interventricular septum. (D,E) The stimulus-to-left ventricular peak time remained consistent at 69 ms during both high- and low-output pacing.

**Figure 2:**
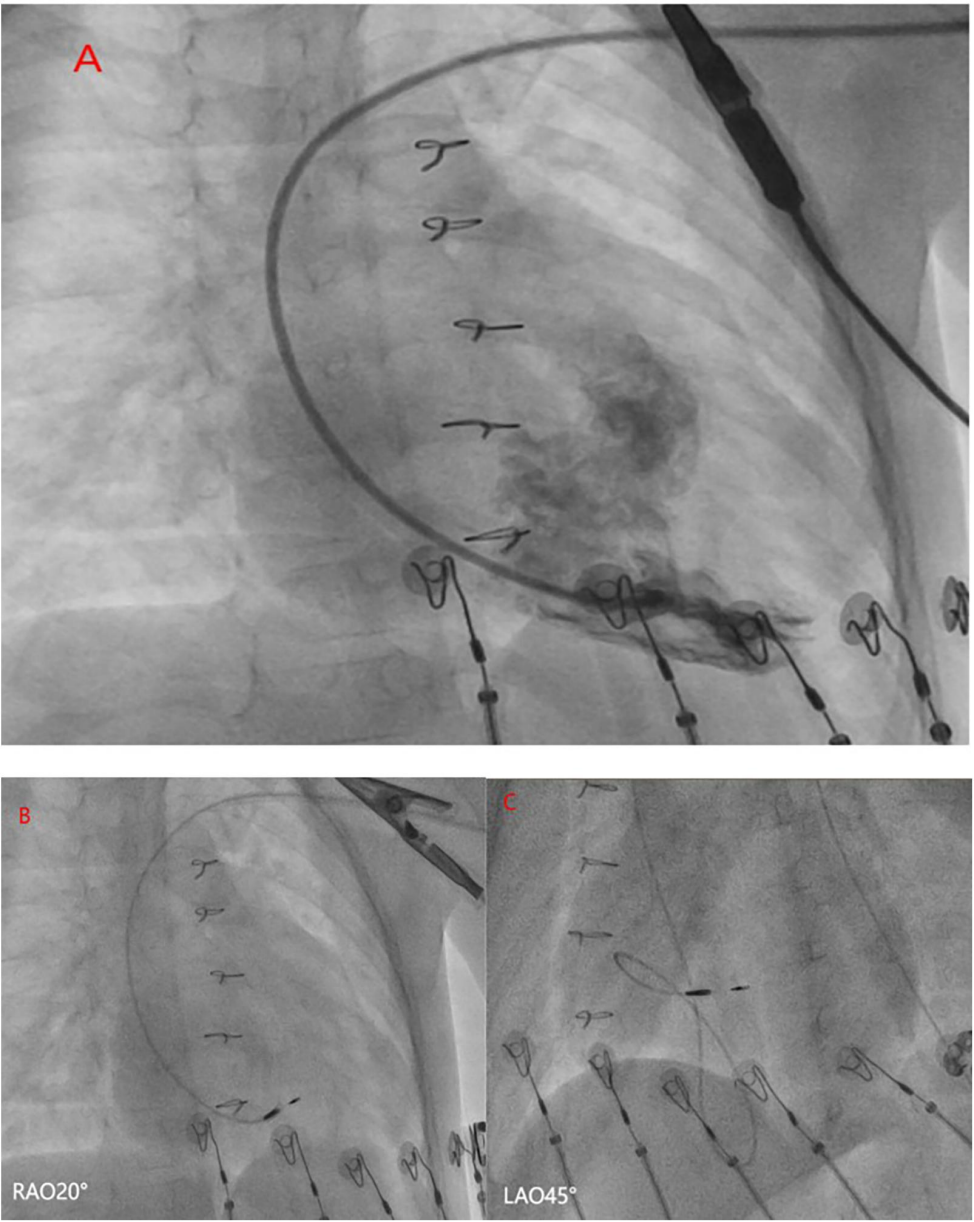
**(**A) Right ventriculography showing the tricuspid annulus. (B,C) Final lead implantation position.

A 3-year-old girl was admitted with bradycardia. She had undergone ventricular septal defect repair with a tissue patch and mitral valve annuloplasty at our hospital approximately 3 years previously. Postoperatively, she developed third-degree atrioventricular block. Electrocardiography revealed third-degree atrioventricular block combined with right bundle branch block (Figure 1A). Echocardiography showed no obvious ventricular-level shunting. Laboratory tests showed no abnormalities. Permanent pacemaker implantation was recommended, and a single-chamber pacemaker was implanted in December 2025. The patient was placed in the supine position and administered intravenous anesthesia. An endotracheal tube was inserted, and mechanical ventilation was initiated. The surgical site was routinely disinfected, and sterile drapes were applied. Under fluoroscopic guidance in the right anterior oblique (RAO) 30° projection, the left axillary vein was punctured using the Seldinger technique, and a guidewire was advanced into the inferior vena cava. Make an incision 1.5 cm below the clavicle to create the pocket. A sheath was delivered via a guidewire and right ventricular angiography was performed to visualize the tricuspid valve annulus (Figure 2A). The apex of this structure was designated as the HIS region. The 3830 lead was inserted into the right ventricular septum via the C315His sheath, and the top of the C315His sheath was moved 1.5 cm anteriorly downward (Figures 2B,C). At this location, the V1 pacing pattern exhibited an rsR′ morphology (Figure 1B). The lead was then rotated to the left ventricular septal surface, during mapping, a sharp left bundle branch potential was observed at the target site (Figure 1C). High- and low-voltage pacing yielded consistent left ventricular activation times, and the V1 pacing pattern exhibited a qR morphology (Figures 1D,E). The paced QRS duration was shorter at the final selective LBBP position compared with that at the superficial right ventricular septal contact site. The pacing site was considered to be in the LBB region. Pacing parameters were measured as follows: S-LBBP threshold 2.5 V; sensitivity 8.0 mV; impedance 723 Ω. The lead was fixed with sutures and connected to a Model 7102 permanent pacemaker. The incision was closed in layers. After 3 months of follow-up, the pacemaker programming parameters were satisfactory, with an LBB pacing threshold of 1.0 V and an impedance of 574 Ω. Echocardiography indicated normal cardiac size, with a left ventricular ejection fraction of 60%.

## Discussion

**Figure 3:**
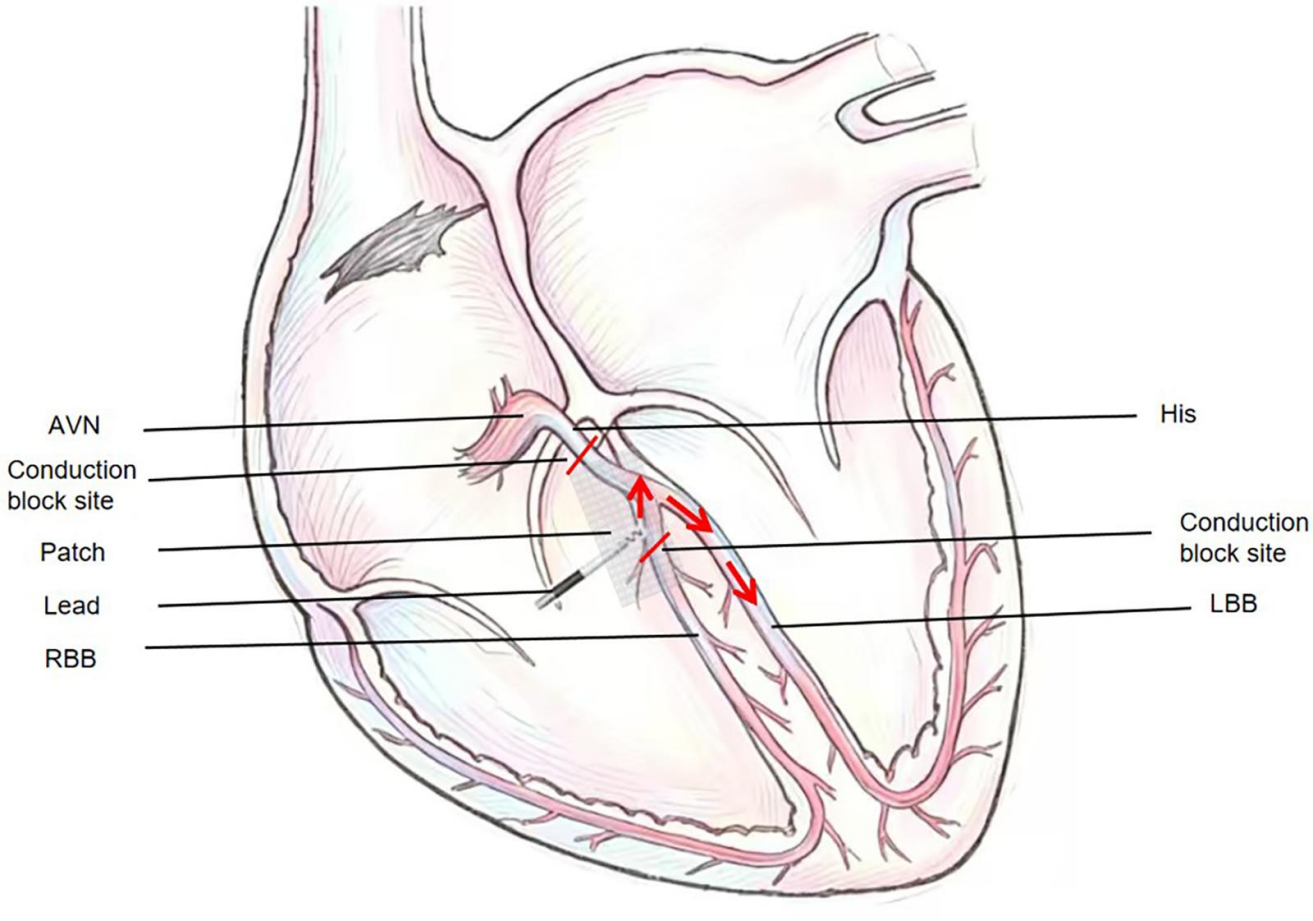
Schematic diagram of the mechanism for RBBB morphology when the lead initially contacts the right ventricular septum.

This case involves a child who developed third-degree atrioventricular block and RBBB following VSD repair. A permanent pacemaker was implanted. During the initial phase of LBBP, the lead contacted only the right ventricular septal myocardium and immediately produced a pacing morphology mimicking RBBB. This phenomenon is exceedingly rare. Pacing at this site typically produces rapid right ventricular activation via the right bundle branch, with delayed left ventricular activation occurring through slow transseptal myocardial conduction, resulting in a LBBB morphology (5, 6). This child underwent patch repair for multiple defects in the membranous portion of the ventricular septum. This anatomical region is a critical site for the bifurcation of the His bundle and the origin of the right bundle branch (7, 8). Surgical patch suturing, local tissue traction, and postoperative scarring may all injure the proximal conduction system in this region. It is hypothesized that when the pacing lead contacts the right ventricular septum, electrical impulses cannot propagate antegradely along the right bundle branch. Instead, impulses may activate the left bundle branch in a retrograde fashion, leading to initial left ventricular depolarization and the resultant RBBB-like pacing morphology (Figure 3). Of note, the ECG manifestations in this child may reflect irreversible structural impairment of the proximal conduction system (His bundle and right bundle branch) secondary to VSD repair (7). In the absence of detailed intracardiac activation mapping, the exact underlying conduction mechanism remains speculative. Notably, intraoperative real-time fluoroscopic monitoring was performed, and the observed pacing pattern was RBBB, which was not attributable to lead mispositioning, myocardial perforation, or left ventricular pacing. Lead perforation was excluded by stable pacing parameters, appropriate lead impedance, and the absence of pericardial effusion or diaphragmatic stimulation on fluoroscopy. Inadvertent left ventricular pacing was ruled out by the lead tip position on the right ventricular side of the septum under RAO 30° fluoroscopic view and the absence of a left ventricular lead trajectory.

The rare electrocardiogram manifestations in this case have significant clinical implications. First, in children with ventricular septal defect who have undergone repair surgery and are subsequently diagnosed with third-degree atrioventricular block, left bundle branch pacing may elicit a right bundle branch block like pattern immediately upon electrode contact with the right ventricular septum. When this occurs, clinicians should prioritize considering structural damage at the proximal right bundle branch or His bundle bifurcation, rather than electrode displacement or myocardial perforation, and avoid misinterpreting the finding as an abnormal procedural event. There is no need for blind electrode repositioning; instead, the pacing site should be comprehensively assessed using intraoperative fluoroscopy, pacing parameters, and intracardiac electrograms (5, 9). Second, during the initial stage of lead implantation, although these patients exhibited right bundle branch block on pacing electrograms, the ventricular activation sequence remained nonphysiological. In contrast, left bundle branch pacing directly stimulates the left bundle branch system, thereby achieving more physiological ventricular activation (9). This study was a single case report. Only three months of clinical follow-up were available in this patient, which is insufficient to evaluate the long-term impacts of left bundle branch pacing on cardiac function and pediatric growth and development. Given the lack of intramural activation mapping, the exact ventricular activation sequence and underlying conduction pathway could not be directly confirmed. As acknowledged herein, longer-term follow-up is still required to further clarify the chronic clinical effects of LBBP in children.

## Data Availability

The raw data supporting the conclusions of this article will be made available by the authors, without undue reservation.
